# Bioavailability of Nutritional Resources From Cells Killed by Oxidation Supports Expansion of Survivors in *Ustilago maydis* Populations

**DOI:** 10.3389/fmicb.2018.00990

**Published:** 2018-05-17

**Authors:** Mira Milisavljevic, Jelena Petkovic, Jelena Samardzic, Milorad Kojic

**Affiliations:** Institute of Molecular Genetics and Genetic Engineering, University of Belgrade, Belgrade, Serbia

**Keywords:** oxidative stress, regrowth, starvation, genome integrity, liquid holding

## Abstract

After heavy exposure of *Ustilago maydis* cells to clastogens, a great increase in viability was observed if the treated cells were kept under starvation conditions. This restitution of viability is based on cell multiplication at the expense of the intracellular compounds freed from the damaged cells. Analysis of the effect of the leaked material on the growth of undamaged cells revealed opposing biological activity, indicating that *U. maydis* must possess cellular mechanisms involved not only in reabsorption of the released compounds from external environment but also in contending with their treatment-induced toxicity. From a screen for mutants defective in the restitution of viability, we identified four genes (*adr1*, *did4*, *kel1*, and *tbp1*) that contribute to the process. The mutants in *did4*, *kel1*, and *tbp1* exhibited sensitivity to different genotoxic agents implying that the gene products are in some overlapping fashion involved in the protection of genome integrity. The genetic determinants identified by our analysis have already been known to play roles in growth regulation, protein turnover, cytoskeleton structure, and transcription. We discuss ecological and evolutionary implications of these results.

## Introduction

One of the enduring appeals of the basidiomycotan fungus *Ustilago maydis* is its resistance to large doses of UV or ionizing radiation so that it has been used as a model for studying DNA repair for many years. In nature, this phytopathogenic microorganism lives a double life. As a dimorphic fungus, it grows as a unicellular yeast in its sporidial saprophytic form but becomes mycelial in the parasitic state after infecting maize tissue. Thus, in its saprophytic stage, it must contend with UV-irradiation, oxidative stress caused by desiccation and rehydration, DNA-damaging toxins produced by soil bacteria and other fungi, whereas in its parasitic stage, it is subjected to the innate immune system of its host. In the response to microbial infections, plants can produce massive amounts of antimicrobial reactive oxygen (Keller et al., 1998) and reactive nitrogen species (Crawford, 2006), so that superoxides, peroxides, and hydroxy radicals generated may cause oxidative stress affecting all cellular macromolecules of the infecting pathogen. Hence, it would seem that *U. maydis* is under powerful environmental pressure to combat deadly genotoxic stresses encountered in both of its life styles. On the other hand, the molecular knowledge of the cellular mechanisms that would have a direct bearing on our understanding of *U. maydis*’ ecological success is still fragmentary. For instance, even regarding the feature that marks *U. maydis* out among other eukaryotes [its extreme resistance to irradiation (Holliday, 1965)], the comprehensive molecular knowledge that would explain this trait is still lacking. Namely, despite the studies aiming at understanding the mechanisms of DNA repair in *U. maydis* that have emerged during the past 50 years and extending from genetics to comparative genomics, the molecular factors and cellular operations that constitute this remarkable capacity still remain enigmatic (Holloman et al., 2008). Nevertheless, it is evident from genetic studies in *U. maydis* that recombinational DNA repair has a central role in radiation resistance since mutation in any one of several genes that control homologous recombination results in extreme sensitivity to UV and gamma-rays; there was a four to five log reduction in survivors at the doses that caused no loss of viability in wild-type strains (Ferguson et al., 1997; Kojic et al., 2002, 2003). Although such powerful recombination repair would obviously contribute to the fitness of saprophytic cell populations, it is interesting that, except for *dss1*, all the other recombination mutants tested were able to proliferate, form galls, and even generate teliospores in planta.

Further, any general comment about the genetic and molecular knowledge that would impact our understanding of the ecological fitness of *U. maydis* cell populations should note that the most of the above studies have primarily used approaches and methods limited to exploring DNA repair and mutagenesis in a replication-dependent manner, i.e., under conditions of relatively rapid growth. Since cell division is a relatively less frequent state for free-living microorganisms (especially when the environment becomes unfavorable), the overall picture of the cellular mechanisms employed by this organism in the maintenance of its genome integrity may be rather one sided. Indeed, we know almost nothing about how this organism might maintain its genome integrity under non-growing (starvation) conditions after strong stress, a situation that might be closely related to what may happen in nature (flooding after a long period of oxidative stress caused by radiation and prolonged bouts of dehydration). Arguably, this could have been a prevailing environmental pressure that shaped adaptation of this organism to arid ecosystems of central America as this is the apparent center of origin for *U. maydis* and its ancestral teosinte hosts (Stukenbrock and McDonald, 2008). In fact, the dominant hypothesis related to the evolution of the extremely radiation-resistant organisms such as the bacterium *Deinococcus radiodurans* and the bdelloid rotifers holds that their extraordinary resistance to oxidative stress could actually be a consequence of their evolutionary adaptation to survive prolonged episodes of desiccation (Gladyshev and Meselson, 2008; Slade and Radman, 2011).

Pondering about these questions and about possible methodological approach for experimental investigation of the recovery from heavy oxidative insults under conditions where lack of nutrients precludes growth, we have decided to entertain the possibility of using liquid holding (LH) as an assay system that might open new venues leading to further insights into molecular players and cellular mechanisms that underlie the ecological and evolutionary fitness of *U. maydis.* The assay essentially consists in monitoring post-stress survival of the treated cells held under non-nutrient/starvation conditions and accordingly, by its inner logic, may be the one modeling the scenario *U. maydis* might periodically encounter in the wild. When used in *Saccharomyces cerevisiae* LH led to discovery that the yeast cells possess a truly remarkable capacity to recover from massive damage if kept in non-growth solutions before plating (Patrick and Haynes, 1964; Patrick et al., 1964). The effect was referred to as LH recovery and it has been extensively investigated in *S. cerevisiae* at genetic level. However, it should be added, this impressive recovery has not been investigated from a molecular biology standpoint so that understanding of the molecular events underlying this phenomenon is utterly limited. On the other hand, the ability of *U. maydis* cells to accomplish LH recovery has never been probed. Therefore, we decided to explore LH recovery in this organism but the analysis of the post-treatment behavior of *U. maydis* cells has revealed that the enhancement of viability, in contrast to *S. cerevisiae*, was not achieved by intracellular repair but by multiplication of the survivors at the expense of the killed cells. This observation was a stimulating point for further investigations so that we have gone on and obtained evidence supporting the existence of cellular operations involved in the recycling of the intracellular compounds released from the killed cells as an ambivalent source of rich but risky nutrients. Further, the insight that was most indicative came from the finding that some of the factors required for regrowth are also involved in the maintenance of the genome integrity.

## Materials and Methods

### *U. maydis* Genetic Methods

Manipulations with *U. maydis*, culture methods, gene transfer procedures, survival after DNA damage by ultraviolet (UV) light, methyl methanesulfonate (MMS), or hydroxyurea (HU) treatment, etc., have been described previously [see (Kojic et al., 2008; de Sena-Tomas et al., 2015) and references therein]. Strain UCM520 (*nar1-1 met1-2 a2 b2*) was used as the nominal wild type for clastogen treatment, and genetic analyses and mutagenesis (Kojic et al., 2002). *met*, *nar*, and *a* and *b* indicate auxotrophic requirement for methionine, inability to utilize nitrate, and mating type loci, respectively. For assessment of growth in suspensions of treated cells UCM520 was marked with resistance to geneticin by random restriction-enzyme (*Bam*HI)-mediated integration (Bölker et al., 1995; Kahmann and Basse, 1999) of a *Bam*HI cassette consisting of the strong constitutive *otef* promoter fused to a neomycin phosphotransferase (*neo*) gene expressing resistance to geneticin (Kojic and Holloman, 2012). Cell counting was performed under a microscope using a hemocytometer. Haploid and diploid *U. maydis* cells were grown in liquid YEPS medium (1% yeast extract, 2% sucrose, and 2% peptone) at 30°C shaking at 200 rpm. Growth rates of UCM520 and mir mutants were calculated from the logarithmic plots of colony forming units (CFU) number data versus time using equation *k* = (log N2-log N1) ^∗^ 2.303/(t2-t1). N1, N2-number of cells in time points t1 and t2, respectively. The results were analyzed by Student’s *t*-test using SPSS statistical software. *P*-values < 0.05 were considered significant.

Following growth, the cells were washed three times with distilled water. For peroxide treatment, 2 × 10^7^ washed cells were suspended in 1 ml of 10 mM Fe^3+^-sodium EDTA. H_2_O_2_ was added to start the Fenton reaction and the suspension held at 30°C. After 10 min cells were collected by centrifugation, washed twice in water, and resuspended in water at 2 × 10^7^/ml and plated immediately on rich (YEPS containing 2% agar) medium to measure survival or else maintained at 30°C with agitation for 24–72 h before plating to measure recovery. Spot assays were performed by making serial 10-fold dilutions from the initial cell suspension of 2 × 10^7^/ml then spotting 10 μl aliquots of each dilution in sequence on solid medium. Plates were incubated for 3 days at 30°C for colonies to develop. For UV treatment, cells were resuspended at 2 × 10^7^/ml in water, irradiated with a 254 nm germicidal UV lamp at dose rate of 2.0 J/m^2^/s in an open Petri dish with gentle swirling. Aliquots were removed and plated immediately to measure survival. Irradiated cells were maintained in water at 30°C with mixing for 24–72 h before plating to measure recovery.

For the time-course leakage studies, 10 ml of suspensions of cells in 10 mM Fe^3+^-sodium EDTA (at a concentration of 2 × 10^7^ cells per ml) was treated with increasing dose of hydrogen peroxide for 10 min at 30°C. Following the peroxide treatment the cell suspensions were washed twice in water, resuspended in water at 2 × 10^7^/ml, and incubated in capped flasks mounted on an oscillating shaker in a 30°C room. At various time points, 1 ml aliquots of the suspensions were withdrawn, the cells were pelleted by centrifugation, and the supernatants were decanted. To insure that the solutions were cell free, the supernatants were passed through millipore filters (0.45 μm) and then immediately examined for 260-nm-light-absorbing compounds in a Ultrospec 3300 *pro* (Amersham Bioscience) Spectrophotometer.

### Mutant Screen and Gene Cloning

Exponentially growing cells of strain UCM520 were spread on solid medium and irradiated with 254 nm UV light to a survival frequency of about 0.05%. Colonies arising from approximately 1200 mutagenized survivors were tested individually as described above for failure to reconstitute viability upon the treatment with 0.4% H_2_O_2_ in the presence of 10 mM Fe^3+^-sodium EDTA. Four candidates with loss of ability to grow were chosen for additional study. The defective genes in mir237, mir354, and mir754 were cloned by complementation of sensitivity to 0.02% MMS after introducing a genomic DNA library prepared in a self-replicating vector with a hygromycin resistance marker as described previously (Kojic et al., 2002). mir107 was cloned by three consecutive rounds of treatment with 10 mM Fe^3+^-sodium EDTA, 0.4% H_2_O_2_, and a 24-h period in water. After each round of treatment, survivors were grown to saturation in medium containing 100 μg/ml hygromycin. 2 × 10^7^ cells were collected and treated again through a subsequent round. After the third round plasmid was extracted from survivors and the sequence analyzed.

The DNA sequences of the termini of the complementing fragments were determined to delineate the bounds of the cloned fragments, which were then matched to the *U. maydis* genome sequence in the annotated Munich Information Center for Protein Sequences (MIPS) database^1^. Obvious candidate genes identified by inspection of the genomic sequences were apparent in all cases. These were confirmed by subcloning to a single open-reading frame and retesting for complementation. This was followed by amplifying the candidate open-reading frame from genomic DNA of the individual mutants using polymerase chain reaction, then determining the DNA sequence to establish identity of the inactivating mutation. Genes and corresponding proteins identified in the mutants have the following *U. maydis*-annotated genome database (UMAG) identifiers: mir107-UMAG_04456 (Adr1); mir237-UMAG_04865 (Did4); mir354-UMAG_15019 (Kel1); and mir754-UMAG_10143 (Tbp1).

## Results

### LH Restitution of Viability in *U. maydis* Results From Cell Multiplication

**FIGURE 1:**
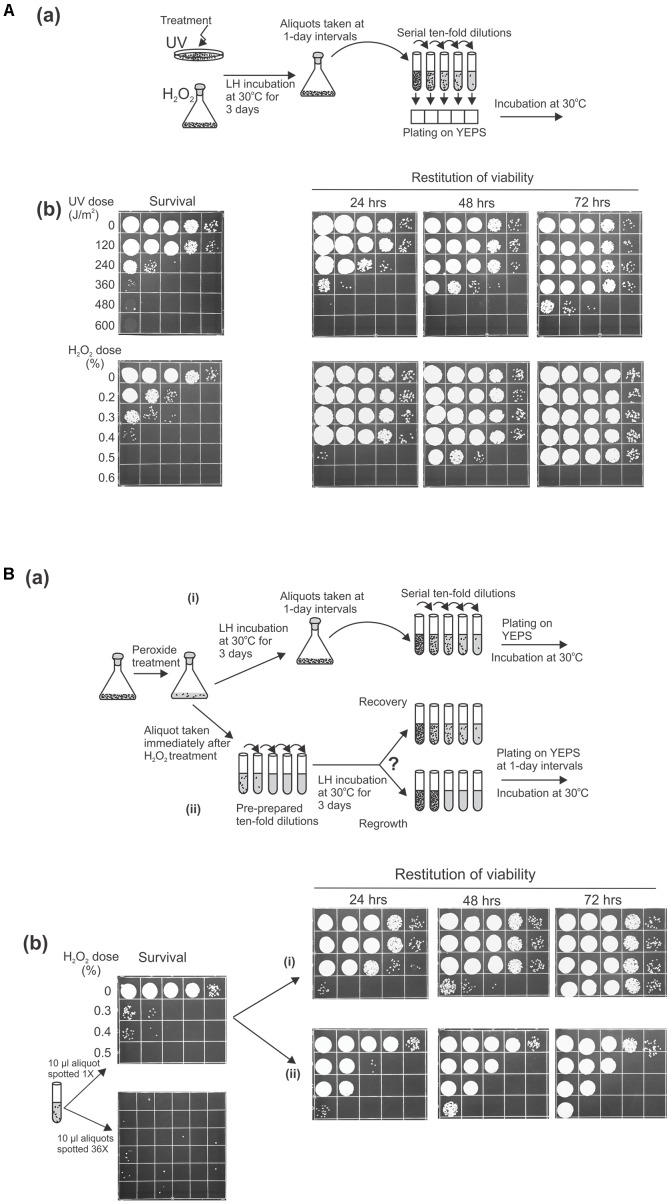
Liquid holding restitution of viability in *Ustilago maydis*. Stationary phase wild-type cells (2 × 10^7^/ml) washed and resuspended in water were used in all experiments. Aliquots (10 μl) of 10-fold serial dilutions were plated on growth medium for determination of cell viability. **(A,** **a)** Schematic diagram of the experimental design. **(b)** Cells irradiated with UV or treated with Fe^3+^-sodium EDTA + H_2_O_2_ (peroxide-treatment) were plated immediately to determine survival, or were held in water for the indicated times before plating. **(B,** **a)** Schematic diagram of the experimental design. **(b)** Peroxide-treated cells were plated immediately (survival). The increase of cell viability was monitored at 1-day intervals in (i) the original suspensions of the treated cells incubated in water at 30°C under continuous agitation before preparation of serial dilution and plating or in (ii) pre-prepared 10-fold serial dilutions, following incubation under the same conditions. All experiments were performed at least three times and representative results are shown.

As an approach that might lead to understanding the means by which microbial cells maintain genome integrity in inhospitable environments, we wanted to probe the role of post-treatment LH on cellular recovery by following the behavior of *U. maydis* cells held in water or dilute phosphate buffer for a prolonged period after being exposed to otherwise lethal doses of UV light or reactive oxygen species (ROS) generated by the Fenton reaction. As an initial examination of genotoxin sensitivity, we treated stationary phase cells grown in rich medium with UV or with Fe^3+^-EDTA and hydrogen peroxide (referred to below as peroxide treatment) to induce severe damage, then measured survival by plating both sets on rich medium. There was the expected increased killing with increasing dose of UV or of peroxide (**Figure 1A**). Then, we tested whether the suspensions of treated cells incubated under nutrient-free conditions over a prolonged period prior to plating could recover from damage (by periodically taking aliquots, making serial 10-fold dilutions, and plating them on complete medium). We observed that with additional time in distilled water (or in the phosphate buffer) at 30°C under continuous agitation the suspensions of previously damaged cells exhibited an astonishing degree of restitution of viability (**Figure 1Ab**). Namely, the number of cells capable of forming visible colonies is markedly increased over that observed on immediate plating. Even the cell suspensions that had been inflicted with such lethal levels of UV or ROS sufficient to reduce survival by five orders of magnitude (on immediate plating) could reconstitute almost the initial level of viability when incubated for 72 h before returning to complete medium.

After these initial insights, it became of immediate and fundamental interest to determine whether this striking restitution of viability was due to intracellular repair of the clastrogen-induced damage or the result of multiplication of the undamaged cells. To distinguish between these possibilities, we design an experiment in which the methodological operation preceding the delayed plating was modified (**Figure 1Ba**). In parallel with periodically taking aliquots from LH suspensions and subsequently making a serial of 10-fold dilutions (to be used for scoring the fraction of viable cells), we also monitored the viability in the pre-prepared 10-fold dilutions that were made immediately after peroxide treatment and incubated under the same conditions. Since the restitution of viability following peroxide treatment appeared faster than after comparable levels of killing by UV, in the following investigations we chose to concentrate our focus on restoration from oxidative damage induced by peroxide treatment, rather than from UV. For this experiment, we treated *U. maydis* cells with the doses of hydrogen peroxide that decreased the surviving fraction to 0.01% (or less) and distributed the suspensions according to the design articulated above. Thus, if the post-treatment restitution of viability is mediated through intracellular repair (true recovery) then the increase of the surviving fraction should in principle be uniformly seen in both series of LH dilutions. However, if the increase of post-treatment viability is the result of cell multiplication then in the series of pre-diluted suspensions the growth that follows the incubation would strictly be evident only in the dilution tubes that contained viable cells at the moment of distribution. As shown in **Figure 1Bb**, the increase of viability was not equivalently present in both branches of the experimental setting [(i) and (ii)]. Indeed, when the pre-prepared 10-fold dilution tubes were incubated at 30°C for 1, 2, or 3 days prior to plating the increase of viability was not found in each tube but only in those that would be predicted to contain surviving cells at the moment of distribution, gauged by the colony count on immediate plating (**Figure 1Bb**). The finding would, thus, strongly suggest that the increase in viability seen during LH reflects the growth and proliferation of the undamaged cells presumably by reabsorption of the intracellular compounds released from the killed cells. In this context, it is also noteworthy that similar results were obtained regardless of the mating type and ploidy of the strains analyzed by the foregoing strategy (data not shown).

### Cells Surviving Oxidative Damage Scavenge Metabolites From Dead Cells to Grow and Reproduce

What enabled cell growth and multiplication during repopulation? Since the post-treatment reconstitution of viability is not achieved by recovery but by proliferation of the surviving subpopulation and because the repopulation does happen without any external supplementation of nutrients, it was logical to assume that the nutrients required for the growth and numerous multiplications of the survivors must be obtained from the killed cells. Therefore, to investigate the bioavailability of the endogenous metabolites, we first examined the release of the intracellular material from cells damaged by oxidation into the suspending medium. Thus, we treated UCM520 cells with increasing dose of hydrogen peroxide (0–0.6%) and, as an indication of leakage of the intracellular compounds, we have chosen to estimate the total nucleotidic content (that should include nucleosides, nucleotides, and other nucleic acid derivatives) of the supernatants by measuring absorbencies at 260 nm. The release of the UV-absorbing (nucleotidic) biomolecules into the extracellular medium was determined over a 6-h period following the treatment.

**FIGURE 2:**
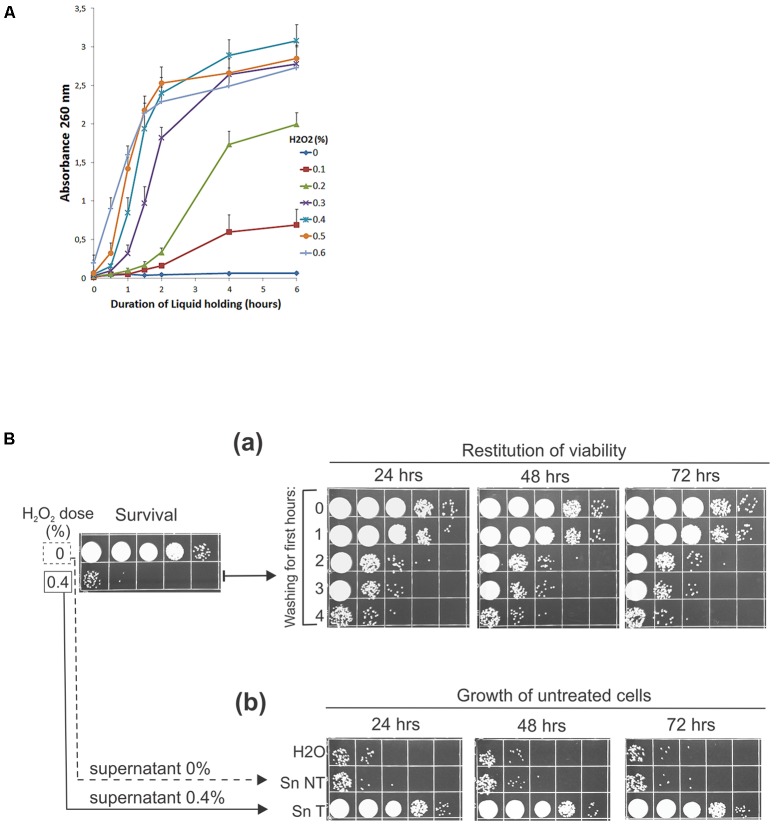
**(A)** Leakage of nucleotidic material from H_2_O_2_-treated cells. **(A)** 2 × 10^7^ wild-type cells/ml were treated with indicated doses of peroxide, washed, and incubated in water for 6 h. Aliquots were taken, centrifuged, and millipore-filtered supernatants were used for measurements of absorbance at 260 nm periodically. The error bars indicate standard deviations of three independent experiments. **(B,** **a)** Peroxide-treated cells were washed at 15-min intervals for the first 0–4 h after the treatment and then incubated in water for the indicated times before spotting. **(b)** 8 × 10^3^ of untreated geneticin-resistant cells/ml were incubated in H_2_O, supernatant of untreated cells (Sn NT), and supernatant derived from peroxide-treated cells (0.4% H_2_O_2_) after 4 h of incubation at 30°C (Sn T). Aliquots were plated on the complete medium containing 100 μg/ml geneticin. All experiments were performed at least three times and representative results are shown.

The time-course curves of the leakage from peroxide treated and untreated cells, graphed in **Figure 2A**, clearly demonstrate that the peroxide treatment does induce release of nucleotidic material into the suspending medium and that the leakage is dose dependent. Little or no leakage is seen in the non-treated control. However, the leakage is increased by peroxide treatment. On the whole, increasing the dosage increases the rate (gauged by the slope of the curves) and total amount of leakage. For the higher doses of peroxide (0.3–0.6%), the leakage was very rapid for approximately 2 h and then ceased. Thus, the peroxide-induced leakage of the intracellular compounds in the treated cell populations may provide an accessible supply of nutrients that would suffice to enable the survivors to keep growing and replenish the population decimated by oxidative damage.

To strengthen this idea further we performed two other kinds of experiments more directly concerned with the ability of living cells to utilize the leaking biomolecules. First, if *U. maydis* cells need to perform recycling to repopulate, the depletion of the leaked cellular material (by periodically washing the treated cells) should reduce the number of cells multiplied during LH incubation. Indeed, when we performed this experiment the increase in surviving fraction was significantly reduced when the treated cells were washed and resuspended in distilled water periodically during the first few hours after peroxide treatment (**Figure 2Ba**). Second, in the reciprocal kind of experiment we asked whether the amount of the released cellular material was sufficient to support as robust growth as seen during LH restitution of viability? Accordingly, we obtained a conditioned medium derived from non-treated and 0.4% H_2_O_2_-treated cell suspensions by centrifuging and filtering out cells through millipore filters following 4 h of incubation under LH conditions. The supernatants were inoculated with a fixed number (8 × 10^3^/ml) of untreated cells. The inoculum prepared in distilled water served as a control. As shown in **Figure 2Bb**, the supernatant derived 4 h after the treatment did contain nutrients in quantities sufficient to support the growth of non-treated cells to the extent large enough to account for the restitution of viability that was seen in the suspensions of treated cells (compare **Figures 2Bb** with **Figure 2Ba**). Furthermore, since only a slight (if any) increase in growth is seen in the supernatant derived from untreated cells, relative to that in the control, we conclude that the treatment-induced leakage was necessary and sufficient to allow the survivors to repopulate. Finally, since UCM520 is a methionine auxotrophic *U. maydis* strain, the above results indicate that the strain can scavenge enough methionine to meet its growth requirement during LH proliferation. As no exogenous methionine was supplied by the medium, the only remaining source of methionine is the dying subpopulation of cells. This is a direct evidence that *U. maydis* cells can scavenge nutrients released by the dying cells during LH starvation. In addition, we have probed a prototrophic *U. maydis* R521 strain under the same condition but found no greater cell proliferation than that obtained in the case of UCM520 indicating that the supply of methionine was not a limiting factor that would determine a maximum of UCM520 growth (data not shown).

For these several reasons, we, therefore, conclude that the enhanced viability seen after the absorption of massive damage and following the incubation of the treated suspensions of *U. maydis* cells for a prolonged period prior to plating is, in contrast to *S. cerevisiae*, achieved through cell multiplication by feeding on the intracellular compounds leaked from the damaged cells. Furthermore, considering the consequences of the treatments, i.e., the leakage of intracellular compounds and by them supported post-treatment regrowth, we can (only) conditionally call this overall phenomenon: “restitution of viability under non-growing condition” or “repopulation under starvation conditions,” meaning specifically that the regrowth is realized without any external supplementation of nutrients. For simplicity, the acronym “RUS” will refer here specifically to post-treatment regrowth (or repopulation) under starvation by recycling nutrients released from the killed cells. The term “recycling” is used in this paper to indicate the overall process of effective utilization of nutrients from dead cells without necessarily specifying the cellular operations involved.

### The Opposing Effect of Cellular Leakage Products on Cell Multiplication

**FIGURE 3:**
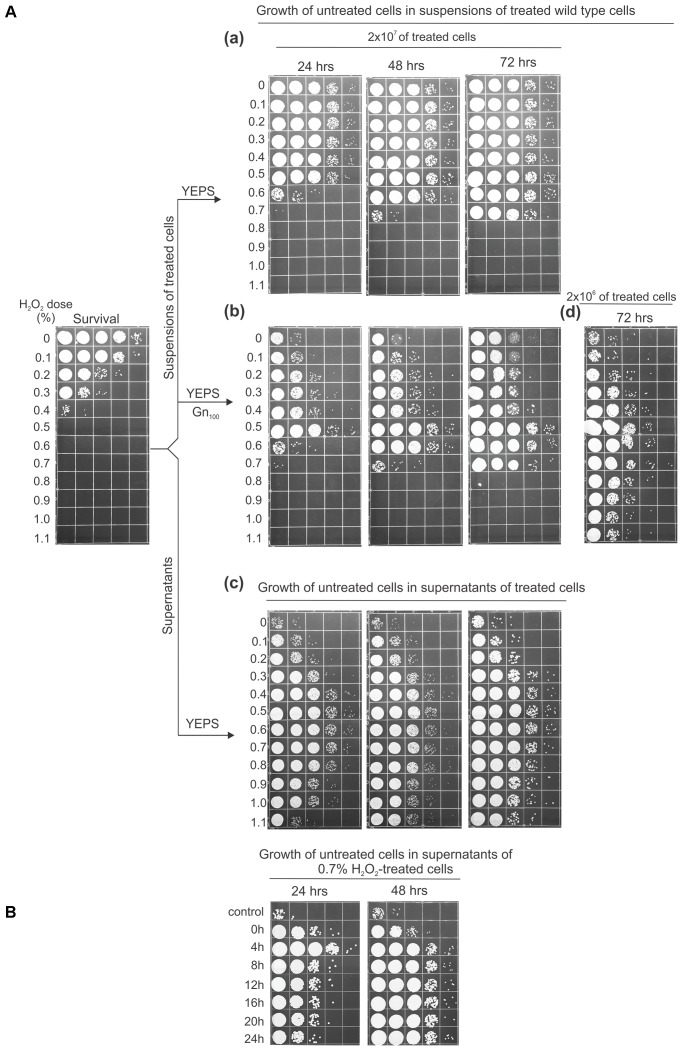
Cell growth effected by the substrates released from peroxide-treated cells. **(A)** To test the effect of the substrates in the suspensions of treated cells, amounts of 8 × 10^3^ untreated geneticin-resistant cells/ml were inoculated into the suspensions of 2 × 10^7^ **(a,b)** or 2 × 10^6^ wild-type cells/ml **(d)** treated with the indicated doses of peroxide and incubated for 3 days in water at 30°C under continuous agitation before serial dilution and plating on YEPS medium **(a)** or YEPS medium containing 100 μg/ml geneticin **(b,d)**. The survival of the treated cells is shown in the far left panel. **(c)** 8 × 10^3^ untreated geneticin-resistant cells/ml were inoculated into the supernatants of 2 × 10^7^ treated cells, incubated as described, and plated on the complete medium. **(B)** Growth of untreated cells (8 × 10^3^/ml) in supernatants of 0.7% H_2_O_2_-treated wild-type cells. Aliquots of supernatants were taken at 4-h intervals during 24-h incubation in water. All experiments were performed at least three times and representative results are shown.

The effect of leaked intracellular material on the proliferation of fresh cells added into the suspensions or supernatants derived from treated cells is shown in **Figure 3**. For this experiment, suspensions of UCM520 cells (at a concentration of 2 × 10^7^ cells/ml) were treated with increasing dose of hydrogen peroxide (between 0 and 1.1%) and the killing was measured by ability to form colonies on immediate plating. Each of the treated cell suspensions was divided in two aliquots, one to be directly inoculated with fresh cells and the other to obtain cell-free supernatants by centrifuging and filtering the suspensions after 4 h of LH incubation. Then, a fixed number (8 × 10^3^) of untreated cells was added to each set of aliquots. To distinguish the proliferation of the untreated cells from the multiplication of the survivors the added strain was a geneticin-resistant UCM520 derivative. The geneticin (GenR) resistance marker (encoded by the *neo* gene) was assumed to be effectively neutral in the absence of drug selection, having, thus, no effect on competitive advantage under these growth conditions. The extent of growth was evaluated after 24, 48, and 72 h of incubation of the suspensions at 30°C under continuous agitation and by plating the cells on YEPS medium containing 100 μg/ml geneticin. Since in the treated cell suspensions the proliferation of the fresh cell inoculums was in competition against the growth of the survivors the sum of the expanding subpopulations was estimated by plating the cells on YEPS medium.

The extent of growth of the untreated cells after 24 h of incubation, both in the suspensions and in the supernatants, was approximately proportional to the peroxide dose up to 0.5% (**Figure 3A**), after which there was a marked (in the suspensions-**Figure 3Ab**) or gradual (in the supernatants-**Figure 3Ac**) decrease suggesting the production and release of the compounds that interfere with cell proliferation. This decrease was overcome after 72 h in the suspensions that were treated with 0.6 and 0.7% peroxide. On the other hand, in the cell suspensions treated with peroxide concentrations >0.7%, not just the absence of growth but even the loss of viability of the untreated cells was evident. However, the negative effect was partially overcome after 72 h of incubation when the suspensions of the treated cells were 10-fold diluted and then inoculated with 8 × 10^3^ of untreated cells (**Figure 3Ad**), indicating that the efficient cell reproduction in a particular suspension of treated cells is a matter of the ratio of treated to untreated cells.

Several other observations regarding the results seen in **Figure 3A** are appropriate to mention. First, as the LH incubation continued the concentrations of cells in the supernatants obtained from heavily treated cell suspensions were progressively increased. Therefore, it seems likely that the lower number of viable cells seen in these supernatants after 24 h of incubation was not due to a reduction of available nutrients but rather because of the elevated levels of toxic molecules. Next, it may be noted that the concentrations of GenR cells grown in the suspensions treated with 0.3 and 0.4% peroxide were appreciably lower relative to those in the corresponding supernatants indicating that the competition between the freshly added cells and the survivors for the limited source of nutrients does occur in these mixtures. However, even though there were no survivors that would compete for the nutrients in the 0.8–1.1% peroxide-treated suspensions the untreated cells still could not proliferate in them, as opposed to their expansion seen in the matching supernatants obtained at 4 h from the same treated cultures. This comparison suggests that the inhibitory compound/s might be released during the later phases of the dying-cells decomposition. To investigate this possibility we treated UCM520 cells with 0.7% peroxide, incubated them for 24 h under LH conditions, and prepared supernatants by taking aliquots at 4 h-intervals. The obtained supernatants were inoculated with fresh cells and incubated as above. The results obtained in this experiment (**Figure 3B**) are of considerable interest in that the cell concentration after 24 h incubation exhibits a significant fall (about 10-fold) in the supernatants derived at 8 h of post-treatment incubation compared to that reached in the 4 h-supernatant. Referring still to the same panel, it is seen that the further falling of the cell concentration is less pronounced so that the cell concentration in the supernatant obtained at 24 h was about 50-fold lower than in the 4 h-supernatant. In accord with previous observations the concentrations of cells became approximately equal as the incubation continued for 48 h suggesting again that the lag seen after 24 h of incubation was not due to a reduced supply of nutrients but more likely because of the toxic molecules produced during progressive degradation of cellular components. Finally, although toxicity is increased in supernatants with the longer LH incubation, the inhibitory effect of the supernatants was not large enough to account for the negative effect seen in the treated cell suspensions. Evidently, the toxicity cannot be completely transferred to the derived cell-free medium. The nature of these processes as well as the chemical nature of the toxic compounds is a problem which at the moment cannot be resolved.

In sum, the experiments have shown that the released intracellular compounds may have opposing biological activity, that increasing the dose of peroxide as well as prolonging the post-treatment incubation increases the toxicity of the suspending medium, and that the toxicity of the released material can be overcome by increasing the ratio of untreated to treated cells or by extending the time of LH incubation. The treatment-induced degradation of cellular components needs further investigation, but in this work we were more interested in the question of how *U. maydis* cells perform recycling of the released material during LH repopulation. Accordingly, we were primarily interested in discovering which cellular factors would be involved in this process.

### A Screen for Mutants Defective in RUS

Motivated by the finding that the leaked cellular compounds are an ambivalent source of nutrients which are nevertheless efficiently used by *U. maydis* cells, we made three simple assumptions that would guide further investigations: (1) *U. maydis* cells must possess and implement cellular mechanisms involved in reabsorption, processing, and reuse of the leaked material in order for the cells to grow and multiply under the LH conditions, (2) the recycling of the released substrates may entirely be carried out by cellular factors and operations employed during regular growth in rich medium, and (3) *U. maydis* might employ specific (unknown) cellular factors required exclusively for the management of the toxic derivatives released from dying cells. Needless to say, there is no *a priori* reason to exclude any of these possibilities. It is certainly conceivable that the processing of the toxic derivatives could, indeed, be carried out entirely by cellular factors principally devoted to “regular” growth. Namely, it could simply be that the factors and operations used in the “normal” growth may have an innate potency to respond to the elevated challenge posed by toxic biomolecules so that the capacity is expressed only during the recycling in the devastated population. On the other hand, it would certainly not be surprising if the *U. maydis* cells possess factors that are implemented exclusively during the detoxification of the leaked cellular material. What makes this particular hypothesis rather provoking is that the analysis of the *U. maydis* genome sequence revealed that more than one-third of genes identified were of unknown function (Kämper et al., 2006), leaving open the intriguing possibility that RUS might be carried out by an exclusive subset of factors that may have been bypassed by research focused primarily on understanding the physiology and dynamics of cells during rapid growth.

**FIGURE 4:**
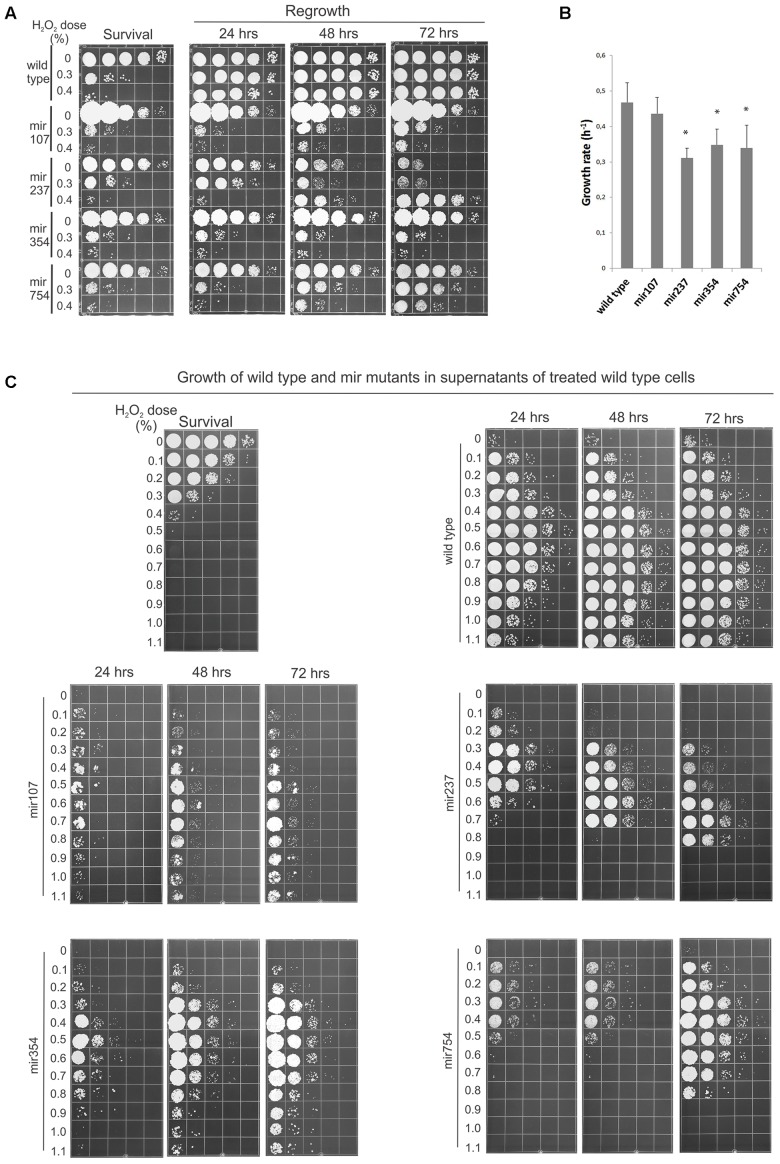
Mutants defective in regrowth under starvation (RUS). **(A)** Cells (2 × 10^7^/ml) from the indicated strains were treated with Fe^3+^-sodium EDTA + H_2_O_2_ and allowed to regrow over a 72 h period in water. Samples (10 μl) were plated immediately to determine survival or were held in water for the indicated times before plating to determine viability. **(B)** Growth rates of wild-type and mir mutants in complete medium (YEPS). The error bars indicate standard deviations. Asterisks indicate significant differences (*p* < 0.05) between growth rates of each mutant and wild type. **(C)** 8 × 10^3^ cells/ml (wild-type and four mir mutants) were inoculated into supernatants of 2 × 10^7^ wild-type cells/ml treated with the indicated doses of peroxide and incubated for 3 days in water at 30°C under continuous agitation before serial dilution and plating on the complete medium. The survival of the treated cells is shown in the upper left panel. Pictures are representative of at least three independent experiments.

Therefore, as an approach for identifying genes underlying the cellular machinery involved in reabsorption and processing of the leaked cellular material we decided to search for mutants defective in the process. Accordingly, we devised a screen in which we mutagenized cells, allowed them to form colonies, and tested these individually for loss of ability to regrow in the suspension of peroxide-treated cells. Of 1200 candidates tested four had particularly compelling phenotypes in RUS activity and were chosen for more depth analysis. Relative to wild-type, all four mir (mutation in RUS) mutants exhibited a significant lagging in terms of regrowth when the peroxide-treated cell suspensions were held in water for 24 h before plating (**Figure 4A**). In the cases of mir107 and mir354 there was little or no restitution of viability even after 72 h of incubation. However, in mir237 and mir754 the regrowth was picking up as the LH incubation continued, although there still remained a lag between mir754 and wild-type strain even after 72 h of LH. mir237 responded to starvation conditions in a different manner. When untreated with peroxide all the mutants were able to maintain viability with high efficiency after a 24-h period of starvation. However, untreated mir237 became increasingly feeble in viability the longer it was held in water, but surprisingly cell multiplication become more robust following 48 h incubation with the more damage the mutant received. We refer to this as the inverted phenotype as it seems to be a mirror image of the survival when plated immediately after damage (**Figure 4**, compare mir237 survival and 72 h panels). In sum, all four mir mutants have shown reduced ability of RUS, but they differed among themselves in the pattern of their post-treatment response.

After these initial characterizations we asked whether the reduced restitution abilities of the mutants reflected only reduced growth rates or also their inability to contend with the treatment-induced toxicity? Accordingly, we first estimated growth rate for wild-type and each mutant growing them in YEPS. As shown in **Figure 4B** all the mutants grew slower than wild type. However, we could not establish a clear correlation between the growth rates of the mutants and their capacity to perform RUS. For example mir107, which could not restitute post-treatment viability even after 72 h of LH and which of all the mutants formed the smallest colonies when incubated on solid YEPS medium, was to our surprise closest to wild type in terms of growth rate in liquid culture. Consequently, in hopes of delineating the underlying impairment in mir mutants we decided to characterize their LH response in more detail by monitoring their behavior in a broader spectrum of challenges. Accordingly, we assayed for the ability of the mir mutants to grow in supernatants obtained from UCM520 cell suspensions (again at a concentration of 2 × 10^7^ cells/ml) treated with increasing dose of hydrogen peroxide (between 0 and 1.1%) as well as in 10-fold diluted suspensions of heavily treated (0.8–1.1%) cells of the same strain. 8 × 10^3^ of untreated wild-type or mutant cells was added to each sample and the growth was examined at 1 day intervals. The results for the growth in the supernatants and in suspensions of treated cells are shown in **Figure 4C** and in Supplementary Figure S1 in the Supplemental Material, respectively. Although each mutant has exhibited a distinctive pattern of its growth behavior, the general conclusion was that all four mutations conferred growth disadvantage for their carries under the challenging conditions. The most severe phenotype was observed in mir107. It was lagging far behind wild type across the entire spectrum of supernatants (**Figure 4B**), and its growth was likewise totally inhibited in all the suspensions of treated cells (Supplementary Figure S1). After 24 h of incubation mir237 showed only a modest reduction in the low dosages supernatants. On the other hand, its growth was dramatically reduced even after 72 h in the high dosages supernatants. As noted above, mir237 was profoundly impaired in its ability to maintain viability the longer it was held in water. The same was true for those supernatants in which mir237 was able to proliferate. Indeed, the pattern of sequential growth and decrease of viability displayed by mir237 in supernatants derived from cultures treated with increasing concentrations of peroxide is truly intriguing. Further studies are required to elucidate reason/s for this puzzling behavior. Compared to each other, mir354 and mir754 showed somewhat similar pattern of growth in the supernatants. However, while mir354 could not grow in the treated cell suspensions at all, mir754 mutant experienced a 1-day lag period before growth could pick up under these conditions. Notably, however, there was no growth of this mutant in the highest dosages supernatants. Taken collectively, two main conclusions can be drawn from these results. First, the growth dynamics of each mutant has its distinct pattern of environmental sensitivity. Second, and more important, since all four mutants exhibited a dramatic inhibition of growth under severe challenge (either in the heavily treated cell suspensions or in the high dosages supernatants, or in both), but under which wild-type cells could still proliferate, we conclude that the cellular factors affected in the mutants all underlie repopulation responses including efficient mitigation of the treatment-induced toxicity.

### Response of Mir Mutants to Genotoxins

**FIGURE 5:**
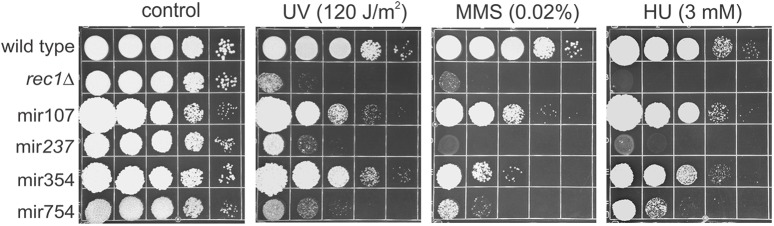
Response of mir mutants to genotoxins. Mutants were plated on solid medium then irradiated with UV or else plated on medium containing MMS or HU. The *rec1*Δ mutant was used as a control to illustrate sensitivity to all three treatments. The testing was performed two times and representative results are shown.

The experimental results reported in the previous sections have dealt with overall cytotoxic challenges posed by the leaked compounds. Since all mir mutants exhibited enhanced sensitivity to the released cellular material, it was of considerable interest to examine sensitivity of the mir mutants to several genotoxins (UV, MMS, and HU) under normal growth conditions (**Figure 5**). Since the testing was also done in hopes of finding a phenotype that could be exploited for gene cloning, for comparison a mutant defective in a DNA damage checkpoint gene (*rec1*Δ) that could be readily cloned by complementing DNA damage sensitivity to any one of several genotoxic agents was included as a reference and control. Perhaps surprisingly, given the approach used for hunting for the mutants, two of them, mir237 and mir754, showed extreme sensitivity to DNA damaging agents and to the DNA replication stressor HU while mir354 exhibited mild sensitivity to MMS. Thus, the analysis indicated a clue concerning the broader context of the cellular role of mir gene products. The findings suggested that at least some of the components of the cellular machinery involved in facilitating *U. maydis* for highly efficient reuse of the leaked intracellular compounds also operate in some important and overlapping fashion in the response to DNA damage and replication stress. In addition, the analysis indicated the possibility of cloning the genes by selection for resistance to MMS.

### Gene Identification

**FIGURE 6:**
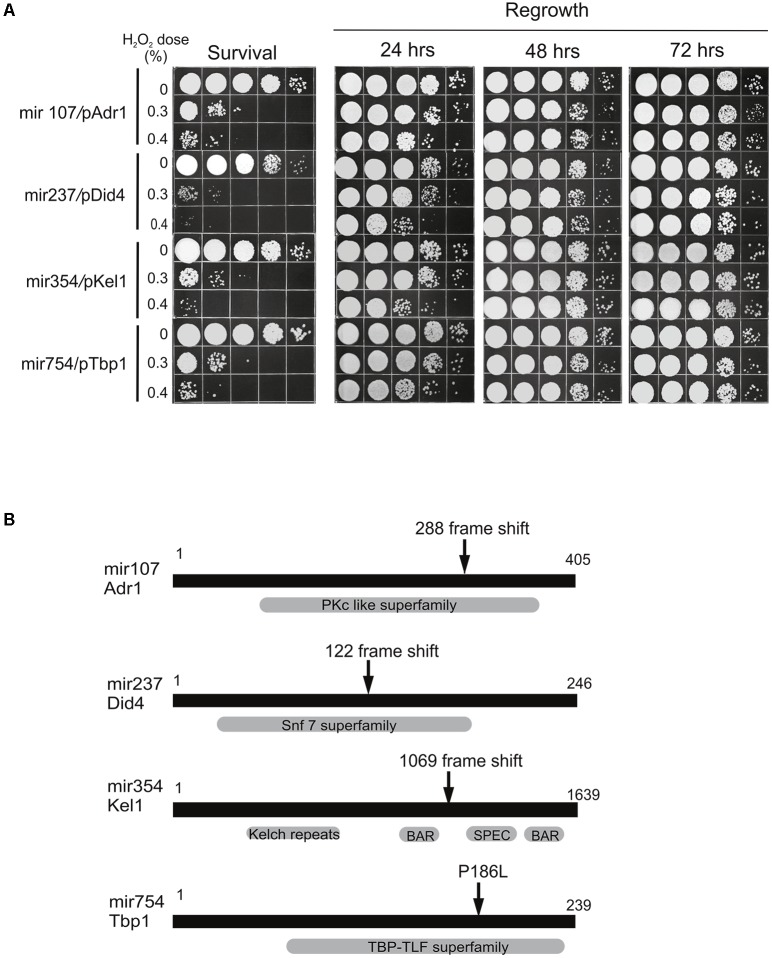
Identification of mir mutants and complementation. **(A)** Cells from the indicated strains transformed with a self-replicating plasmid containing the cloned gene were treated with Fe^3+^-sodium EDTA + H_2_O_2_ and allowed to regrow over a 72-h period. All experiments were performed at least three times and representative results are shown. **(B)** Domain architecture of proteins identified from the screen for RUS mutants is shown schematically in dark gray with amino acid residue indicated. Protein classification and conserved domains are shown in light gray. Protein identifiers in the MIPS-annotated database are as follows: UMAG_04456 – Adr1; UMAG_04865 – Did4; UMAG_15019 – Kel1; and UMAG_10143 – Tbp1. Arrows indicate the point of mutation determined in the mutant gene.

With the exception of mir107, the mutants displayed sensitivity to MMS with a differential from wild-type large enough to enable cloning by complementation. Therefore, we introduced a genomic library prepared in a self-replicating plasmid vector into the three MMS sensitive mutants (mir237, mir354, and mir754) and screened transformants for candidates that were resistant to killing by MMS. Plasmid DNA was extracted from likely candidates and genes present were determined from the sequence. Candidates were subcloned when necessary to narrow down the complementing activity to a single open-reading frame (**Figure 6A**). We recovered plasmids from all three mutants that complemented the mutant phenotype. To verify that the cloned genes represented the wild-type version of alleles present in the three mir strains and not a bypass suppressor, the sequences of the alleles in the mutants were determined and in each case an inactivating mutation was identified (**Figure 6B**).

Cloning mir107 was more challenging because there was no obvious genotoxin sensitivity phenotype associated with this strain. Therefore, a different approach was taken. Here we opted for three consecutive rounds of peroxide treatment and RUS restitution of viability. First, the genomic library was introduced into mir107. Then all transformants were collected, expanded through one round of growth, then treated with peroxide, and allowed to reconstitute the viability under starvation conditions. Cells harboring a complementing DNA fragment would preferentially regrow after peroxide treatment. The process was repeated twice more to enrich the population for the complemented cells. After the final round of enrichment randomly chosen individual clones were tested for RUS restitution after peroxide treatment and two of six were positive. These contained the identical cloned DNA fragment.

The genes identified were attributable as follows: mir107 is defective in the gene encoding a 405 residue homolog of protein kinase A (PKA) catalytic subunit Adr1, a regulator of cell proliferation in response to nutrients (Dürrenberger et al., 1998; Broach, 2012). The mutant allele results from a frame shift at codon 288 resulting in a C-terminal truncation deleted of residues that comprise part of the active site. mir237 is defective in the gene encoding a homolog of Did4 a non-essential protein of 246 amino acids involved in vacuolar protein sorting (Vps) (Amerik et al., 2000). The mutant allele results from a frame shift at codon 122 causing a C-terminal truncation. mir354 is defective in the gene encoding a 1639 amino acid homolog of Kel1, a non-essential protein involved in cell fusion and morphology and exit from mitosis (Philips and Herskowitz, 1998). The mutant allele results from a frame shift to produce a variant protein of 1069 amino acid residues. mir754 is defective in the gene encoding a homolog of TATA-box-binding protein Tbp1, an essential 239 amino acid general transcription factor that functions in assembly of pre-initiation complexes for RNA polymerases (Cormack and Struhl, 1992). The mutant allele contains a missense mutation resulting in a P186L amino acid change that could potentially alter the TFIIB interaction surface and DNA interaction surface.

## Discussion

Three primary conclusions can be drawn from this study. First, after heavy exposure of a population of *U. maydis* cells to clastogens a great increase in viability is observed if the treated cells are incubated for prolonged period in distilled water. This restitution of viability results from cell multiplication of survivors by feeding on the intracellular compounds leaked from the damaged cells. Second, from a screen for mutants defective in the restitution of viability, we identified four cellular factors that contribute to the process. These factors have been known from previous studies to be dedicated to cAMP-dependent signaling, protein turnover, cytoskeleton remodeling, and transcription. Third, some of these cellular operations required for recycling of the damaged intracellular compounds are also involved in protection of the *U. maydis* genome against various genotoxins such as UV, MMS, and HU.

In this study, we set out to probe a means by which microbial cells might maintain genome integrity in inhospitable environments using post-treatment LH as an assay system. In earlier observations on recovery in viability of *S. cerevisiae* after exposure to radiation or radiomimetic agents, it was found that growth was antagonistic to recovery and that the viability of irradiated cells increased if cells were incubated for extended times in the dark in nutrient-free medium prior to plating (Patrick and Haynes, 1964). Patrick et al. (1964) reported that the post-treatment increase in viability following delayed plating was not attributable to cell multiplication but rather to true recovery as the intracellular repair of the treatment-induced damage. The effect was referred to as LH recovery and it was also supported by fluctuation test analyses (Korogodin, 1993). Curiously, LH recovery in yeast was found to be specific for diploids (Patrick et al., 1964) and variously reported as dependent on Rad51 and Rad52 (Petin and Kabakova, 1981) or independent of Rad52 (Moore et al., 2000). Disparities in strain backgrounds, growth, media, and recovery conditions likely contributed to the differences noted. Studies in haploid and diploid *S. pombe* strains revealed a surprisingly opposite effect, i.e., decreased survival as a result of post-irradiation LH in a non-nutrient medium (Harm and Haefner, 1968; Shahin et al., 1973). Interestingly, the loss of viability of irradiated cells when deprived of nutrients before plating had a positive correlation with the doses used in the treatment, i.e., the more pronounced decrease in post-treatment survival for increasing dose of irradiation was reported (Harm and Haefner, 1968; Shahin et al., 1973). In *U. maydis*, as described above, the findings provide evidence for a differing conclusion: in contrast to the findings in *S. pombe*, after the absorption of massive damage the fraction of viable cells is indeed greatly increased (**Figure 1A**) and, in contrast to *S. cerevisiae*, the enhanced viability is achieved not by intracellular repair but by cell multiplication at the expense of the intracellular compounds leaked from the damaged cells (**Figure 1B**). In considering these differences, it is tempting to speculate that the post-treatment loss of viability in *S. pombe* could actually be a consequence of the poisoning effect of the damaged and leaked intracellular material. If true, the possibility would thus emphasize the competitive importance and ecological advantage of *U. maydis* based on the existence of the cellular mechanisms that enable not only survival in the presence but even the utilization of potentially harmful cellular waste.

Release of the intracellular material outside of the harmed cells has been abundantly investigated in different model systems and in the context of damage inflicted by irradiation (Swenson, 1960), hydrogen peroxide (Brunk et al., 1995), ethanol (Salgueiro et al., 1988), hydrostatic pressure (Shimada et al., 1993), freezing (Sinskey and Silverman, 1970), electroporation (Aronsson et al., 2005). The present study of the leakage phenomenon in *U. maydis* has shown that cells rendered non-viable by peroxide treatment leak a dose-dependent amounts of intracellular substances into the suspending medium. However, the emphasis of this work was not on detailed characterization of the effectiveness of peroxide treatment regarding rates and extents of the cellular leakage nor on precise determination of the chemical nature of the released products but rather upon the effect of the leaked material on the growth of undamaged cells. Accordingly, we found that the peroxide-induced leakage material possessed opposing biological activity. Starting from lower concentrations of peroxide the released material supported growth but at the higher concentrations the released material exerted inhibitory effect that could partially be overcome by extending time of incubation or by increasing the ratio of untreated to treated cells (**Figure 3**). The observation, thus, not only confirmed that *U. maydis* cells could reabsorb the leaked cellular material from their external environment but also suggested that they must possess some means by which the toxic compounds are processed.

What is the molecular basis of such means? Stimulated by the finding that the damaged and released cytoplasmic compounds are an ambivalent source of nutrients, and making the assumption that *U. maydis* cells must possess cellular machinery involved in reabsorption and processing of the leaked material, we performed a hunt for mutants defective in these cellular functions. As a result, we identified four determinants (Adr1, Did4, Kel1, and Tbp1) involved in RUS thus providing initial molecular insights into the cellular operations underlying the process in *U. maydis* (**Figures 4**, **6**). In light of this, we may glimpse something of the complexity and diversity of the cellular mechanism underlying RUS. Namely, the genetic determinants identified by our analysis have already been known to play roles in growth regulation, protein turnover, cytoskeleton structure, and transcription. Furthermore, the mutants in *did4* and *tbp1* exhibited extreme sensitivity to DNA damaging agents and to the DNA replication stressor HU while the mutation in *kel1* showed only mild sensitivity to MMS (**Figure 5**). These finding are of special note because they are most suggestive of the possibility that at least some of the cellular factors involved in management of the leaked intracellular compounds do also operate in some principal and overlapping manner in the response to DNA damage and replication stress. Indeed, this stands to reason, since it would be expected that some of the cellular operations that contributed to contending with treatment-damaged material would be processing genotoxic components as, for instance, damaged nucleotides.

Mutant mir107, defective in Adr1, fails to reconstitute viability yet has no obvious phenotype after being challenged with UV or genotoxins under growth conditions. PKA draws from multiple highly related and redundant catalytic subunits to serve as a master regulator of growth and morphogenesis, to control stress response genes and entry into the quiescent state (Lee et al., 2003; Gray et al., 2004; Broach, 2012; De Virgilio, 2012). We interpret this to mean that the role of Adr1 is removed from the actual mechanics of the recycling but likely is more aligned with signaling and control of RUS. The question of whether Adr1, as a master regulator of growth, exerts its RUS controlling function through common or RUS-specific factors is open to future investigation.

Mutant mir237 showed compromised regrowth kinetics and also is highly sensitive to genotoxin exposure during proliferation. As MMS and HU exert their effects during S phase, it would appear that a consequence of the mutation is stalled replication fork progression. The gene product responsible, Did4, is an ESCRT-III complex factor (Amerik et al., 2000). ESCRT complexes are involved in vesicular trafficking and in destruction of damaged proteins. A number of ESCRT components and associated factors have been identified in yeast in screens for loss of resistance to bleomycin and related compound zeocin (Aouida et al., 2004; Krol et al., 2015). These metal-containing chemoenzymes attack by a free radical-driven process similar to ionizing radiation and Fenton reaction chemistry introducing oxidative lesions in DNA and other cellular components. Other screens in yeast for sensitivity to measures that introduce DNA breakage such as overexpression of a cleavage complex mimetic variant of Topoisomerase I (Reid et al., 2011) or exposure to CtdB toxin (Kitagawa et al., 2007) have also yielded mutants in Vps. The role of Vps factors in repair is not well appreciated nor understood. It could be imagined that Vps participation in removal of damaged proteins might be a necessary prerequisite for rebuilding chromatin and the arsenal of needed DNA repair enzymes. With regard to the disturbed RUS exhibited by mir237, it is tempting to speculate that the slowing down of the rate of regrowth might actually result from compromised processing of the recaptured peptides derived from killed cells. This intriguing hypothesis would, thus, implicate the ESCRT complexes in the recycling of the reabsorbed peptides, a possibility that calls for a systematic and detailed analysis.

The near-complete failure of RUS in mir354 during 72 h incubation implies that the deficiency in the mutant is inside the realm of a crucial cellular operation involved in utilization of stress-released substrates. However, given that Kel1 is a less well-studied cellular factor it is rather premature to propose any strong hypothesis that would suggest a precise role for Kel1 in the recycling activity. Nevertheless, as Kel1 may associate with actin cables (Gould et al., 2014) its function in RUS might involve reorganization of cytoskeleton or vesicle movement or the proper response to nutrient limitation (Care et al., 2004). Thus, only additional research will tell us why Kel1 is so critical for RUS.

Mutant mir754 is sensitive to genotoxins during active growth and very slow in RUS indicating the affected gene function contributes to DNA repair and to the harnessing of the metabolites from killed cells. The gene product Tbp1 is an essential transcription factor that functions in assembly of pre-initiation complexes (Cormack and Struhl, 1992). It is certainly possible that Tbp1’s contribution is in promoting expression of DNA repair factors. Furthermore, emerging evidence reveals possibilities of more direct roles for transcription factors in repair of DNA double-strand breaks perhaps by giving rise to non-coding RNAs that regulate repair (Lee et al., 2009; Chowdhury et al., 2013; Wei et al., 2015; Ohle et al., 2016), by altering chromatin structure (Bonetti et al., 2013) or even by promoting synthesis of transcripts that could serve as templates enabling repair (Keskin et al., 2014). However, the link between DNA repair and RUS activity that was strongly suggested by the phenotype of mir754 mutant cannot be grasped with any plausibility at this stage of investigation. Nonetheless, the mutant is interesting and should be explored further.

An important implication of the above discussion is that the identified genes are involved in a number of diverse cellular processes and that additional studies are required to more fully elucidate the respective roles of each of the above factors in the recycling activity. Also, the picture of the cellular network already seems to be complex, emphasizing the need to identify additional players, including those performing actual mechanics of nutrient acquisition, processing, and utilization of these resources. The hunt for mutants defective in RUS activity has already proved itself to be a promising system for the identification of the cellular factors involved in the process. Therefore, additional mutants will be sought to help define the mechanistic basis of the very processing of the reabsorbed material.

Finally, it is perhaps fitting to conclude this discussion by considering some ecological and evolutionary implications of the above results. As a free-living microorganism and biotrophic fungus infecting maize, *U. maydis* confronts a powerful environmental challenge to combat genotoxic stresses encountered in both of its life styles. Owing to its impressive DNA repair system populations of *U. maydis* cells subjected to severe stress could certainly be, to a great extent, rescued by recovery as an intracellular effect of repair of the harmed DNA. However, the work described here would suggest or emphasize the need to recognize the importance of the cellular mechanism required for the recycling of dead biomass as an important determinant of population rescue from very severe stress. The value of this cellular function for successful population dynamics of *U. maydis* cells would be particularly pronounced in an environment with limited nutrients and under competition from other microorganisms. Clearly, this should potentiate the adaptive advantage/usefulness of the extraordinary resistance of *U. maydis* cells to DNA damaging insults since it could enable the fungus for nutritional benefit and reproduction at the expense of the comparatively less resistant microorganisms in its ecosystem. Indeed, in the nutritionally poor environments those microorganisms able to sustain stress and scavenge nutrients from the dead biomass would have a selective growth advantage. In sum, the mechanism can be seen as an adaptation that broadens fitness since it provides *U. maydis* a capability of sustained growth particularly under massive environmental stress that would cause elimination if the facility were not present.

## Conclusion

Like many other initial studies, this one too provides more questions than answers. What is the cause of leakage induced by peroxide treatment? Does the cell death (by criterion of the ability to form colonies) mean that all cell functions are stopped? What is the chemical nature of the RUS inhibitory chemical/s? Are the identified RUS determinants involved in overcoming oxidative pressure to which *U. maydis* is exposed during its parasitic growth in the corn tissue? Does this organism possess cellular factors that are exclusively used for processing of the toxic derivatives released from dying cells? Answering these and similar questions will require a lot of future work. Our present analysis of mir mutants in *U. maydis* identified four cellular factors that are required for effective growth on the metabolites derived from damaged cells and whose role coincide in part with maintenance of genome integrity. Future studies will extend these initial insights and more fully assess the specific factors and mechanisms involved in recognition, uptake, and efficient processing of the leaked cytoplasmic compounds. Knowing the factors that convert the released cellular material into an apt resource for successful regrowth would be important for understanding dynamics of *U. maydis* cell populations under the environmental challenges that decimate them.

## Author Contributions

MK and MM conceived and initiated the project; designed the experiments; and wrote the paper. MM, MK, JP, and JS performed the research and analyzed the data.

## Conflict of Interest Statement

The authors declare that the research was conducted in the absence of any commercial or financial relationships that could be construed as a potential conflict of interest. The reviewer RW and handling Editor declared their shared affiliation.

## Footnotes

1 http://pedant.helmholtz-muenchen.de/
