# Supplementary material for: Bioavailability of Nutritional Resources From Cells Killed by Oxidation Supports Expansion of Survivors in Ustilago maydis Populations
Source: Front Microbiol. 2018 May 17;9:990. doi: 10.3389/fmicb.2018.00990 (PMC5967202; doi:10.3389/fmicb.2018.00990)
Supplement: Supplementary file 1 [file Data_Sheet_1.DOCX]

**Bioavailability of nutritional resources from cells killed by oxidation supports expansion of survivors in *Ustilago maydis* populations**

Mira Milisavljevic^1^, Jelena Petkovic^1^, Jelena Samardzic^1^, and Milorad Kojic^1^*

^1^Institute of Molecular Genetics and Genetic Engineering, University of Belgrade, Republic of Serbia (IMGGE), Vojvode Stepe 444a, POBox23, 11010 Belgrade, Serbia

Running title: Regrowth under starvation

*Correspondence:

Dr. Milorad Kojic

mdkojic@imgge.bg.ac.rs

Keywords: oxidative stress, regrowth, starvation, genome integrity, liquid holding

**Figure S1. Growth of wild type and mir mutants in the suspensions of treated cells.** 8 x 10^3^ cells per ml (wild type and four mir mutants) were inoculated into 10x diluted suspensions of 2 x 10^7^ wild type cells per ml treated with the indicated doses of peroxide and incubated for 3 days in water at 30°C under continuous agitation before serial dilution and plating on the complete medium.
